# Synthesis and Properties of Poly(Isothianaphthene Methine)s with Chiral Alkyl Chain

**DOI:** 10.3390/ma5020317

**Published:** 2012-02-16

**Authors:** Yu Innami, Hirotsugu Kawashima, Rafaël H. L. Kiebooms, Hideaki Aizawa, Kiyoto Matsuishi, Hiromasa Goto

**Affiliations:** 1 Institute of Materials Science, Graduate School of Pure and Applied Sciences, University of Tsukuba, Tsukuba, Ibaraki 305-8573, Japan; E-Mails: s-yinnami@ims.tsukuba.ac.jp (Y.I.); s-hkawashima@ims.tsukuba.ac.jp (H.K.); aizawa@bunko2.bk.tsukuba.ac.jp (H.A.); kiyoto@bk.tsukuba.ac.jp (K.M.); 2 European Patent Office, Patentlaan 3-9, 2288 EE The Hague, The Netherlands; E-Mail: rkiebooms@epo.org

**Keywords:** π-conjugated polymer, low-bandgap polymer, isothianaphthene, chiroptical activity

## Abstract

We synthesized poly(isothianaphthene methine)s with chiral alkyl chains in the substituent. Resultant polymers are soluble in THF and CHCl_3_. Structure of the polymers was characterized with FT-IR, FT-Raman, and UV-Vis-NIR optical absorption spectroscopy. They showed low-bandgap both in solution and in a form of film. Optical activity of the polymers was confirmed with optical rotatory dispersion. Doping effects on the polymer were also examined with UV-Vis-NIR spectroscopy and ESR measurement.

## 1. Introduction

In the search for new materials for plastic electronics, π-conjugated oligomers and polymers have received much recent interest. These materials are used in applications such as transistors [1,2,3], photovoltaic cells [4,5], molecular wires [6], electrochromic devices [7,8,9], and fluorescence sensors or imaging [10,11,12]. Chiral π-conjugated polymers show optical absorption and chiroptical activity in visible range [13]. Although natural materials such as cellulose and proteins show intense chiroptical activity in UV region (short wavelengths), they show no absorptions at the visible range. On the other hand, π-conjugated polymers have chromophore and show optical functionality in the visible region. The function of optical rotation in visible-near-infrared region can be useful as an optical isolator for optical fibers.

Furthermore, chiral π-conjugated polymers can show a helical structure. Helical structure is generally observed in natural polymers, such as DNA and starch. Synthesis of chiral π-conjugated polymers may lead to mimetic technology of natural polymers.

π-Conjugated polymers have been also studied as emission materials for application of 3D displays and backlights of liquid crystal displays [14]. Low-bandgap π-conjugated polymers have optical absorption at near-infrared region, and show good response to external stimulus [15,16,17]. A structure of poly(arylene methine)s is a candidate for low-bandgap polymers [18,19].

Isothianaphthene (benzo[*c*]thiophene) has attractive electric and optical properties [20,21,22,23]. Isothianaphthene is also well known as a monomer for low-bandgap polymers [24,25]. Polyisothianaphthene derivatives having functional groups such as long alkyl chains, liquid crystalline groups, and radicals were studied [26,27,28]. The isothianaphthene polymers could be materials for electrical and optical devices [29,30]. Combinations of polyisothianaphthene and poly(arylene methine) structures can afford improved characteristics. Additionally, introduction of chiral alkyl side chains for poly(arylene methine) can improve solubility, and provide a function of optical rotation at long wavelengths. Chiral π-stacking can be expected in the form of cast films. In this study, we report synthesis of poly(isothianaphthene methine)s with novel chiral alkyl chains and their optical, chiroptical, as well as doping effects.

## 2. Experimental Section

### 2.1. Technique

Chemicals were purchased from Tokyo Chemical Industry and Wako Pure Chemical Industries. 1,4-Dioxane was used after distillation and the other reagents were used as received. NMR spectra were obtained by using a JEOL JNM-ECS 400 spectrometer. The NMR were measured in chloroform-d (CDCl_3_) and listed in ppm from TMS as the internal standard. Specific optical rotation of chiral compounds was measured in THF with a JASCO P-1010 polarimeter. Fourier transform infrared spectra (FT-IR) of the polymers were taken with a JASCO FTIR 300 spectrometer by the KBr method. Fourier transform Raman (FT-Raman) spectra were obtained by using a Nicolet NXR FT-Raman spectrometer with 1,064 nm excitation wavelength. Ultraviolet-visible-near-infrared (UV-Vis-NIR) absorption spectroscopy measurements were carried out at room temperature by using a JASCO V-630 UV-Vis optical absorption spectrometer and a SHIMADZU UV-3100PC spectrometer with a quartz cell. Optical rotation dispersion (ORD) measurements were performed using a JASCO J-720 spectrometer with an ORDE-307W ORD unit. Molecular weights of the polymers were determined by gel permeation chromatography (GPC) using a JASCO HPLC 870-UV detector with THF as the solvent, with the instrument calibrated with polystyrene standards. ESR measurement of the solid polymer sample packed into a 5 mm quartz tube was carried out with a JEOL JES TE-200 spectrometer.

### 2.2. Monomer Synthesis

**Scheme 1:**
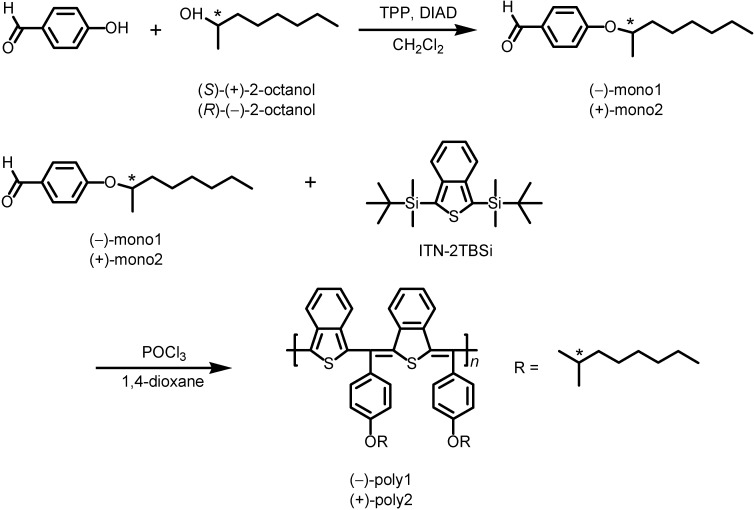
Synthetic routes to poly(isothianaphthene methine)s with chiral side chains. DIAD = diisopropyl azodicarboxylate.

Isothianaphthene monomer, 1,3-bis(*tert*-butyldimethylsilyl)isothianaphthene (abbreviated as ITN-2TBSi) was synthesized in three steps, according to the previously reported method by Okuda *et al.* [31]. Benzaldehyde monomers with chiral side chains were synthesized by the Mitsunobu reaction, as shown in Scheme 1. The monomer ((−)-mono and (+)-mono2) having chiral alkyl group can be obtained in one step.

Reaction of a chiral alcohol with *p*-hydroxybenzaldehyde has been previously reported [32]. Triphenylphosphine (TPP) (2.79 g, 10.65 mmol) was added to a solution of *p*-hydroxybenzaldehyde (1.00 g, 8.18 mmol) and (*S*)-(+)-2-octanol (1.17 g, 9.02 mmol) in dichloromethane (16.00 mL). The mixture was cooled to 0 °C, then diisopropyl azodicarboxylate (DIAD) (40% in toluene, 5.17 mL, 9.82 mmol) was added dropwise. The reaction solution was stirred overnight at room temperature. The resultant mixture was subsequently extracted with dichloromethane, brine and water. The organic layer was dried over magnesium sulfate and filtered. The crude product was purified with column chromatography (silica gel, dichloromethane). Vacuum drying affords (–)-mono1 (Y = 50.7%). The (+)-mono2 was also obtained under the same conditions as synthesis of (–)-mono1 using (*R*)-(–)-2-octanol (Y = 48.7%).

(–)-Mono1: ^1^H NMR (400 MHz, CDCl_3_, *δ* from TMS): *δ* 9.85 (s, 1H), 7.80 (d, 2H, *J* = 8.8 Hz), 6.96 (d, 2H, *J* = 8.8 Hz), 4.47 (q, 1H, *J* = 6.0 Hz), 1.77–1.28 (m, 15H), 0.88 (t, 3H, *J* = 6.4 Hz). ^13^C NMR (100 MHz, CDCl_3_): *δ* 190.5, 163.4, 131.9, 129.4, 115.5, 74.2, 36.2, 31.7, 29.1, 25.3, 22.5, 19.5, 14.0. [α]^20^_D_ = −1.39 deg∙cm^2^∙g^–1^ (THF).

(+)-Mono2: ^1^H NMR (400 MHz, CDCl_3_, *δ* from TMS): *δ* 9.86 (s, 1H), 7.81 (d, 2H, *J* = 8.8 Hz), 6.96 (d, 2H, *J* = 8.4 Hz), 4.48 (q, 1H, *J* = 5.6 Hz), 1.76–1.28 (m, 15H), 0.88 (t, 3H, *J* = 6.8 Hz). ^13^C NMR (100 MHz, CDCl_3_): *δ* 190.7, 163.5, 132.0, 129.5, 115.5, 74.3, 36.3, 31.7, 29.2, 25.4, 22.6, 19.6, 14.1. [α]^20^_D_ = +1.31 deg∙cm^2^·g^–1^ (THF).

### 2.3. Polymer Synthesis

Poly(isothianaphthene methine)s were synthesized with polycondensation between the ITN monomer (ITN-2TBSi) and the benzaldehyde monomer ((−)-mono1 or (+)-mono2) under oxidative conditions. ITN having no protection group at the β-position can be polymerized under oxidative conditions solely. Therefore, *tert*-butyldimethylsilyl groups were introduced onto β-position of the ITN monomer for preventing from polymerization of the ITN solely in the appropriate condition.

A solution of ITN monomer (0.11 g, 0.30 mmol) and (–)-mono1 or (+)-mono2 (0.07 g, 0.30 mmol) in 1,4-dioxane (2.40 mL) was refluxed at 85 °C in the presence of phosphoryl chloride (0.60 mL) for 24 h. Then, the solution was poured into 100 mL of methanol and the precipitate was collected to obtain a dark indigotic blue solid followed by vacuum drying ((–)-poly1; Y = 23.8%, (+)-poly2; Y = 30.5%). The polymers were subsequently neutralized with an excess amount of triethylamine. The polymer (as prepared) was treated with excess amount of triethylamine for obtaining neutral (dedoping) state of the polymer followed by vacuum drying to remove residual triethylamine in the polymer.

The polymerization results are summarized in Table 1. The (–)-poly1 and (+)-poly2 were obtained in the yield of 23.8% and 30.5%, respectively. The yields is comparable to synthetic yield of poly(isothianaphthene methine) having hexyloxy side chains [33]. The number-average molecular weights (*M*_n_) of both (–)-poly1 and (+)-poly2 were estimated to be 2650, the weight-average molecular weights (*M*_w_) were 3030 and 3130, respectively, evaluated by GPC against a polystyrene standard. These molecular weights were slightly higher than that of poly(isothianaphthene methine)s bearing di-*tert*-butylphenol substituents [28].

**Table 1:** Polymerization and optical absorption spectra results.

Polymer	*M*_n_^[a]^	*M*_w_^[a]^	*M*_w_/*M*_n_	*λ*_max_(solution) (nm)	*λ*_max_(film) (nm)	*E*_g_^[b]^ (eV)
**(–)-poly1**	2650	3030	1.1	602	614	1.26
**(+)-poly2**	2650	3130	1.2	601	612	1.26

^[a]^ Polystyrene standard, THF as solvent; ^[b]^ Estimated from onsets of optical absorption spectra in solution.

## 3. Result and Discussion

### 3.1. FT-IR and FT-Raman Spectroscopy

**Figure 1:**
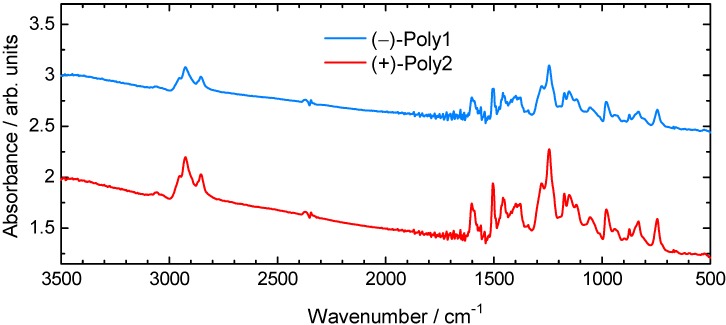
FT-IR spectra of (–)-poly1 and (+)-poly2.

The FT-IR absorption spectra of (–)-poly1 and (+)-poly2 are shown in Figure 1. Intense absorption bands corresponding to C–O–C as an ether stretching were observed at 1,245 cm^–1^ for (–)-poly1 and 1,244 cm^–1^ for (+)-poly2, respectively. The polymers show absorption bands at 1,602 cm^–1^ due to C=C vibrations of a quinonoid isothianaphthene unit [34].

**Figure 2:**
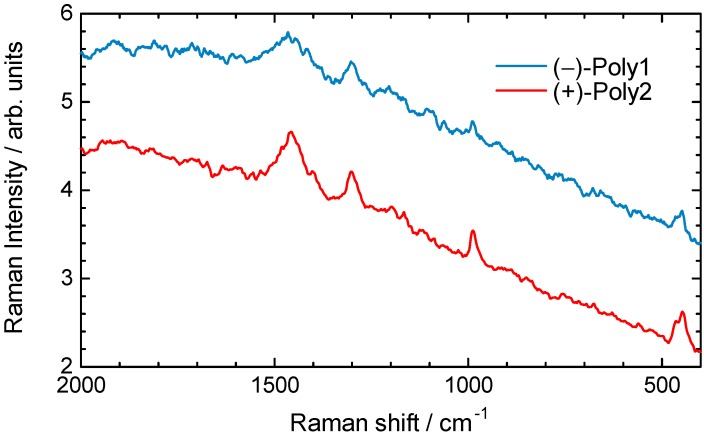
FT-Raman spectra of (–)-poly1 and (+)-poly2.

Figure 2 shows FT-Raman spectra of (–)-poly1 and (+)-poly2. The Raman bands of (–)-poly1 were mainly observed at 1,465, 1,303, 991 and 449 cm^–1^. According to the FT-Raman spectroscopic studies for poly(isothianaphthene methine)s in the previous study, these Raman bands were associated with vibrations of benzenoid and quinonoid isothianaphthene units [28,34]. (+)-Poly2 also shows Raman bands at 1,460, 1,303, 989 and 447 cm^–1^. The results of FT-IR and FT-Raman spectroscopy indicated that (–)-poly1 and (+)-poly2 constructed poly(isothianaphthene methine) units.

### 3.2. UV-Vis-NIR Absorption Spectroscopy

**Figure 3:**
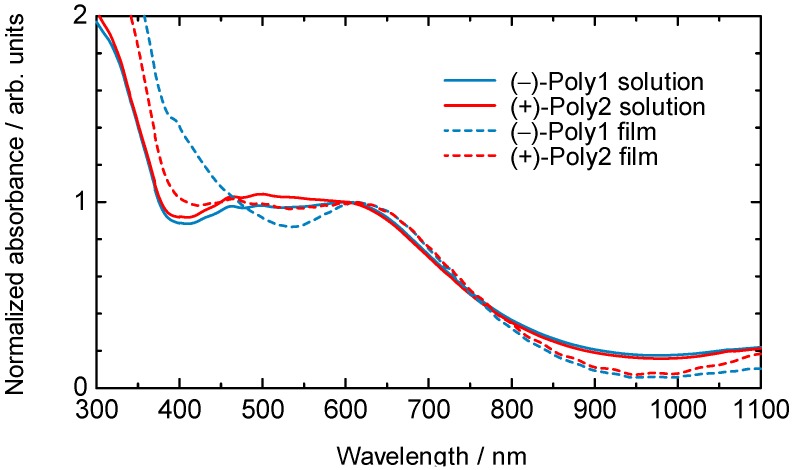
UV-Vis-NIR absorption spectra of (–)-poly1 and (+)-poly2 in solution (0.03 mM in THF) (solid line) and cast films (dashed line).

Figure 3 shows UV-vis-NIR absorption spectra of (–)-poly1 and (+)-poly2 in THF solution, and in the form of cast films. The polymers showed an absorption maximum (*λ*_max_) at around 600 nm in solution state. In comparison with the polymers in solution state, *λ*_max_ of the polymer cast films on a quartz glass showed red shifts by 12 nm for (–)-poly1 and 11 nm for (+)-poly2. From the band-edge of the spectra of both polymers in THF solution, the bandgap (*E*_g_) can be estimated to be 1.26 eV. *λ*_max_ and *E*_g_ of the polymers are listed in Table 1.

**Figure 4:**
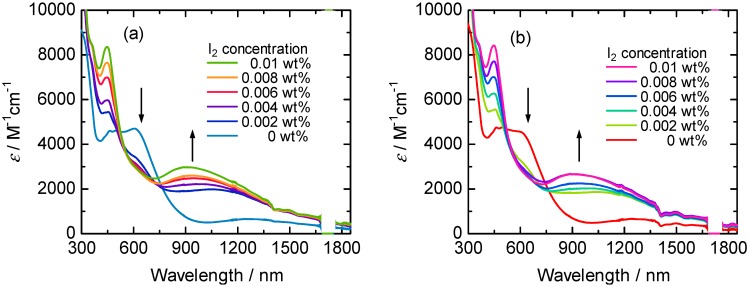
UV-Vis-NIR absorption spectra of (–)-poly1 (**a**) and (+)-poly2 (**b**) in THF solution with iodine doping.

Figure 4 shows UV-Vis-NIR absorption spectra of the polymers in THF solution with iodine doping. An absorption peak at around 600 nm attributed to π-π* transition of the conjugated main chain showed decrease with increasing iodine concentration. At the same time, an absorption peak at around 1,000 nm due to the doping band appeared for the polymers.

### 3.3. ORD Spectroscopy

**Figure 5:**
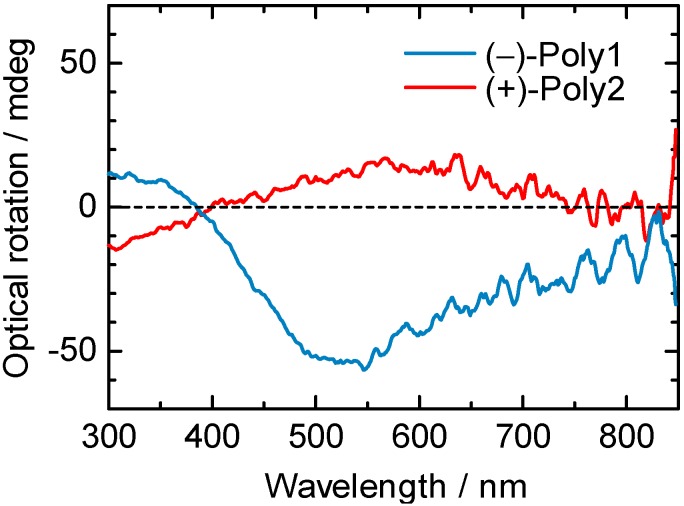
ORD spectra of (–)-poly1 and (+)-poly2 in cast films.

Figure 5 shows ORD spectra of (–)-poly1 and (+)-poly2. Optical rotations at visible range were observed in the ORD spectra of the polymers. (–)-Poly1 polymerized from (–)-mono1 showed a negative optical rotation around 560 nm, and (–)-mono1 also showed a negative optical rotation at 589 nm. (+)-Poly2 gave the same sign of optical rotation as the monomer. These results indicate that the optical rotations at around 560 nm of the polymers are derived from the chiral side chains, suggesting that the sign of optical rotation of the polymers depends on chirality of the side chains.

In the previous research, other types of poly(isothianaphthene methine)s with chiral side chains were reported [26]. However, in this case, poly(isothianaphthene methine)s with a chiral side chain showed no chiroptical activity in visible range. Moreover, poly(isothianaphthene methine)s bearing ferroelectric liquid crystalline groups with chiral center in the terminal displayed no chiroptical activity in the visible range. This is due to the fact that the asymmetric center of the polymers in the side chain has distance from the main chains. In the present study, the asymmetric centers of (–)-poly1 and (+)-poly2 are directly connected to ether oxygen in the substituent, resulting the polymers show chiroptical activity in the visible range.

### 3.4. ESR Measurements

**Figure 6:**
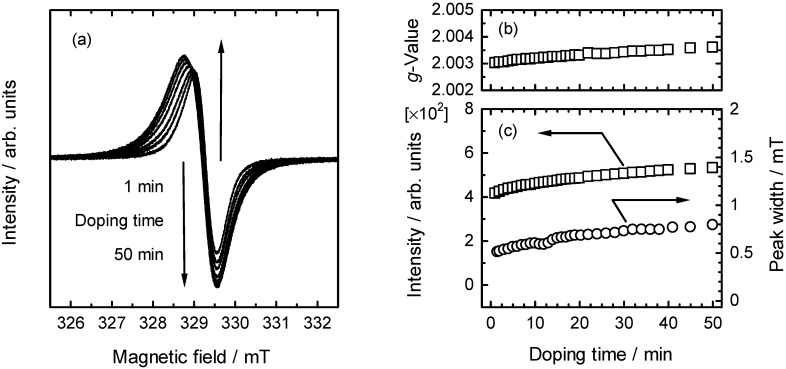
(**a**) ESR spectra of (–)-poly1 in bulk state with vapor phase iodine doping; (**b**) g-Value; (**c**) intensity, and peak width of (–)-poly1 with vapor phase iodine doping for 0–50 min.

ESR measurements were carried out on a polymer bulk sample with vapor phase iodine doping. Figure 6 shows ESR spectra of (–)-poly1 with in-situ doping for 0–50 min. The ESR signal indicates that radical cations were generated along the polymer main chain. The generation of radical cations can enhance electrical conductivity of the polymer [25]. *g*-Value, intensity, and peak-width plots *vs*. the doping time of (–)-poly1 are shown in Figure 6b,c.

The signal intensity gradually increased with the doping time, indicating increase of spin concentration with doping time. However, *g*-value and peak width also increased with doping. Neugebauer *et al.* [34] reported occurrence of strong charge carrier localization with doping confirmed with IR spectroscopic and electrochemical analysis for PITN-methine. The increase of the peak width in the ESR measurements indicates that charge carriers (polarons (radical cations) and bipolarons (dications)) generated by the iodine doping may be localized.

## 4. Conclusions

Chiral π-conjugated polymers can be an important issue in the field of materials science from the view point of comparing basic science to bio-polymers. Industrial applications are expected for opto-electronic organic materials.

We synthesized low-bandgap polymers with chiral side chains under oxidative conditions. An introduction of alkyl side chains allows solubility of (−)-poly1 and (+)-poly2 in organic solvents such as THF or CHCl_3_. Bandgap of the polymers is estimated to be 1.26 eV evaluated from optical absorption spectroscopy. Optical rotations of the polymers were identified in film state with ORD spectroscopy. Furthermore, the polymers showed complementary mirror-image optical rotations. This can be a first example of the synthesis of poly(isothianaphthene methine)s with chiral side chains showing chiroptical activity in the visible range. Doping and dedoping of an electron acceptor for the polymers may afford tuning of optical activity. The polymers may be applied for optical sensing devices by combining the function of optically active low-bandgap character with dopable properties.
